# Innovative Post-Processing for Complex Geometries and Inner Parts of 3D-Printed AlSi10Mg Devices

**DOI:** 10.3390/ma16217040

**Published:** 2023-11-04

**Authors:** Martí Calvet, Anna Domènech, Sergi Vilaró, Toni Meseguer, Lorenzo Bautista

**Affiliations:** Surface Chemistry Area, Applied Chemistry & Materials Department, Leitat Technological Center, 08225 Terrassa, Barcelona, Spain; adomenech@leitat.org (A.D.); svilaro@leitat.org (S.V.); ameseguer@leitat.org (T.M.); lbautista@leitat.org (L.B.)

**Keywords:** post-process, chemical polishing, roughness, additive manufacturing, 3D printing, selective laser melting

## Abstract

A new technology consisting of new and sustainable chemical polishing treatment for aluminum components with complex shapes, such as heat exchangers, manifolds, busbars, aerospace devices, etc., manufactured by Additive Manufacturing (AM) technologies is described in this paper. This technology will contribute to the development of a more efficient manufacturing process driven by AM, reinforcing the main idea of AM, which is based on reducing the amount of material and achieving cost savings through smart and improved designs. The present study shows a significant reduction in the surface roughness of consolidated AlSi10Mg metal parts manufactured by the SLM technique after carrying out the new chemical polishing post-process investigated in this work. Roughness values have been measured by mechanical and optical profilometry. The results obtained demonstrate the effectiveness of the chemical polishing, decreasing the roughness by up to 40%, being a reproducible and repeatable post-process. The presence of smut as solid residues on such types of chemical treatments has been also analyzed with XRF and ICP-MS techniques. The results obtained show that Si and Mg precipitates are removed from the metal surface at the last step of the investigated post-process. The percentages of the elements decrease from 25.0% to 8.09% Si and from 0.86% to 0.42% Mg, achieving the alloy smut-free composition on the metal surface. Tensile strength measurements have shown that the post-process described not only maintains the mechanical properties of the bulk material but, in comparison with non-post-processed parts, a slight improvement is observed with respect to the initial values, Young modulus (61.1 GPa to final 62.2 GPa), yield strength (from 236.8 to 246.7 MPa), and tensile strength (from 371.9 to 382.5 MPa) is observed, suggesting that the post-process has positive impact on the printed metal part.

## 1. Introduction

After iron and steel, aluminum alloys are the most widely used structural materials for automotive, aerospace, and aeronautic applications, among other industries, thanks to their high strength-to-weight ratio, good corrosion resistance, excellent electrical and thermal conductivity, and recyclability. As a result, the additive manufacturing industry has been focused on these areas for many years. The possibility to design and manufacture new optimized geometries using less material has a huge impact on reducing the weight of structural metal parts, which play a big role in fuel consumption in transport applications and, therefore, on cost savings [1,2].

Engineering parts made of aluminum alloys using traditional manufacturing processes includes casting, forging, extrusion, and powder metallurgy. The selected process depends on the final application and the nature of the alloy itself, with 2XXX (Al-Cu or Al-Cu-Mg), 6XXX (Al-Mg-Si), and 7XXX (Al-Zn-Mg) being the commercial Al alloy series that can be strengthened by various heat treatments, while the non-heat-treatable Al series include 1XXX, 3XXX, and 4XXX containing only Si, and 5XXX alloys being those whose strength is increased by mechanical deformation processes [3,4]. These traditional processes do not allow the production of 3D complex structures. Therefore, new manufacturing routes have recently been developed to consolidate complex metal parts, such as Powder Bed Fusion (PBF) [5,6], Directed Energy Deposition (DED) [7], Sheet Lamination (SL) [8,9], Binder Jetting (BJ) [10], and Cold Spray (CS) [11]. Of the powder bed fusion techniques, Selective Laser Melting/Sintering (SLM/SLS) has become the most promising one. This manufacturing technique consists of a powder bed that is melted layer by layer in a protective atmosphere using a high-energy laser beam, and then this molten metal solidifies rapidly [12]. It allows parts to be produced without the need for tooling and appears to be revolutionary, significantly reducing design time and cost. However, conventional aluminum alloys used in traditional manufacturing are neither suitable for laser melting nor for sintering; there are some difficulties overcome such as: a low absorptivity and a high reflectivity to the laser beam, a high cooling rate, an aluminum oxide layer easily formed on aluminum surfaces, the fluctuation of low-melting-point alloying elements as Mg, a high thermal expansion, a high moisture absorption, and poor fluidity due to the formation of powder agglomerates. A detailed study has therefore been undertaken to provide an ideal aluminum powder for SLM technology that meets all these requirements. At present, casting-grade Al-Si alloys with good weldability due to a near eutectic composition, especially AlSi10Mg, have been a key finding to fulfil the conditions required for laser beam manufacturing processes [13,14,15]. Even so, SLS/SLM technique has a major drawback that has already been mentioned: a poor surface quality that does not meet the requirements for industrial applications.

While the focus for Additive Manufacturing (AM) has been put on designing, postprocessing steps of great importance have also resulted. The implementation of AM parts has been more challenging than expected due to the need to remove supports and reduce the high roughness typically obtained after AM processing. After support removal, surface finishing has been identified as the top trend in the AM technological sector for 2022. In fact, nowadays, post-printing processes represent almost 30% of the total project budget related to manufacturing of AM parts [16]. One of the current more challenging demands is not only to improve the AM surface finishing of external surfaces but also to reduce surface roughness of AM inner parts, pipes, and complex shapes. Surface roughness can have a critical effect on properties such as fatigue, strength, friction, and heat transfer [14,17]. It is also of great importance for the joining of metal parts, where excessive roughness can lead to the failure of a compact part or cause corrosion issues [13,18]. In addition, the final finish of a part has a major impact on its cost from the customer’s point of view, representing up to 40% of its market value [19].

Therefore, it can be concluded that it is necessary to implement post-processes which provide a substantial improvement of the final finishing for AM metal parts, not only to improve the appearance of the metal parts but also to guarantee a long service life that better fulfils its function.

External parts of AM end products have been successfully surface-treated by various technologies, with abrasive processes being the most widely used. Vibratory finishing has been successfully implemented in the industry because of its simplicity. It does not require a fixed geometry tool. It is clean and demands little manual labor intervention. AM parts with highly complex shapes are placed in a vessel where fine particles of abrasives are used to achieve a smoothing of the surface by the impacts caused by vibration [20,21,22,23,24]. Compressive residual stresses can occur during the mechanical process and may eventually form the so-called Beilby layer, which contains a mixture of oxides of the base metal and the compounds used for polishing; therefore, the physicochemical properties of the surface layer obtained by mechanical polishing can be different depending on the nature of the underlying alloy, causing mechanical stress which, under certain conditions, can give rise to processes of corrosion [25]. Mechanical post-processes such as vibratory finishing present a potential clogging of abrasive particles particularly for AM inner parts and complex geometries. Surface post-processing of this type of AM parts with complex geometries is still a challenge.

In addition to mechanical finishing, chemical treatments such as alkaline etching, acid pickling, and electropolishing can be used to tackle unevenness of the metallic surface, without altering the bulk properties of the workpiece. Aluminum pitting can be used for surface processing where localized corrosion of the metal is used as an advantage to create uniform surface roughness [26]. Electropolishing is an alternative surface treatment in which the metal to be polished acts as an anode in an electrolytic cell and dissolves. The micro- and macroprojections, or high points of the rough surface, just as regions with burrs, are areas of higher current density than the rest of the surface and dissolve at a faster rate, giving rise to smoother, more level, and/or burred surface. This is why electropolishing is mainly used to achieve mirror finishing quality. The difficulty lies in polishing conducts, cavities, pipes, and those regions shielded by a complex geometry, which are common for AM parts [27].

An alternative post-processing method to improve the surface quality of 3D-printed samples is chemical polishing. In contrast to electropolishing, the lack of current during this process results in a uniform attack all over the surface, avoiding the intrinsic shortcomings of electropolishing on treating cavities and areas with higher current density [28]. Nevertheless, the chemical polishing described to date is very aggressive and uses strong acids to reduce the surface roughness of the object to be polished. Polishing in acid medium is carried out at high temperatures and is not suitable because too much material is removed from the part, and surface defects (pitting) are then created [29].

On the other hand, all the chemical methods described above must face the formation of a smut layer as a by-product of the process. This smut layer for aluminum alloys is primarily composed of an amorphous aluminum hydroxide that can have a significant impact on the resulting morphology of the final surface [26]. In addition, in magnesium- and silicon-containing aluminum alloys (AlSi10Mg being the ones used for 3D printing), coarse constituent Mg_2_Si intermetallic particles and fine Mg_2_Si precipitates that are commonly formed during casting and processing of aluminum alloys can further interfere in the polishing step by forming Mg(OH)_2_ and SiO_2_·xH_2_O sublayers that are particularly difficult to eliminate [30]. As such, removing smut is a key step in order to obtain a functional polished aluminum piece.

According to the needs outlined above, Leitat has recently developed an innovative post-processing method that is particularly useful for complex geometries and inner parts of aluminum 3D-printed metal parts obtained by means of additive manufacturing [19]. This method combines a series of steps which end up with a smut-free polished metal part. First, the metal surface is activated and sensitized to be prepared for the following steps. Secondly, the roughness is faced and reduced in a controlled manner by selective etching of the peaks. Next, the smut formed during the overall process is removed, and, finally, the methodology is repeated *n* times to achieve the roughness required for the end case. All the steps are carried out by immersion in aqueous solutions to guarantee a homogeneous attack to the metal part. With less processing time, less residues, and lower temperatures, this process also contributes to reducing the environmental footprint.

In this study, a novel post-processing method to face complex shapes and inner parts of AlSi10Mg devices consolidated by SLM technique is presented. It is an environmentally friendly post-process involving a low risk for operators and easy automation, that is presented as an alternative to mechanical, electropolishing, and other chemical polishing processes [31,32,33]. Promising results have been obtained in this work, which allow us to envisage a successful future implementation at the industrial level.

## 2. Materials and Methods

Aluminum samples were obtained from selective laser melting technique (SLM) using a Renishaw AM 500 M metal additive manufacturing machine (Renishaw Iberica S.A.U., Barcelona, Spain), spot size 80 µm, layer thickness 60 µm, scanning rotation 67° and argon atmosphere. Al powder was obtained from Renishaw AlSi10Mg commercial powder (Renishaw Ibérica S.A.U., Barcelona, Spain)with the following composition (in wt %) 9.00 to 11.00 Si, 0.25 to 0.45 Mg, <0.25 Fe, <0.20 N, <0.2 O, <0.15 Ti, <0.10 Zn, <0.10 Mn, <0.05 Ni, <0.05 Cu, <0.02 Pb, <0.02 Sn, Al balance, with a layer thickness of 25 µm and 400 W of laser power [34].

### 2.1. Surface Treatment Experimental Part

**Figure 1:**
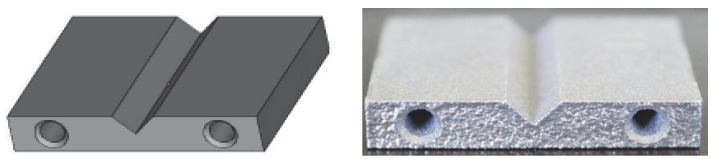
AlSi10Mg metal parts design for post-processing tests; on the left is a schematic design, on the right is a picture of the consolidated metal part.

For this study, several replicates of AlSi10Mg samples were consolidated by SLM to ensure the reproducibility and repeatability of the test. In order to test the efficiency of the post-treatment, a preliminary complex geometry was designed with two pipes and edges and corners to check that they are not rounded during the process (Figure 1).

**Figure 2:**
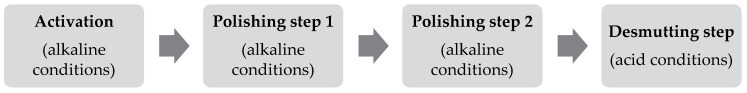
Schematic representation of steps followed on polishing AlSi10Mg using patent WO 2021/094641 [19].

After checking the initial roughness of the samples described previously, the samples were post-processed following the chemical polishing method described in WO 2021/094641 [19]. Activation of the surface is performed under alkaline conditions using sodium hydroxide; then, two polishing steps are performed also by means of an alkaline solution combining sodium hydroxide (1.5%) and potassium carbonate (25%), and finally, the desmutting step is performed using acetic acid (5%). The overall process is performed at room temperature and can be repeated by *n* cycles depending on the initial roughness of the metal parts and the final requirements. The schematic process is represented in Figure 2.

The procedure illustrated in Figure 2 was repeated 8 times according to the initial values from the aluminum samples and the knowledge acquired working with this alloy in previous projects.

A KLA-TENCOR ALPHA STEP D 600 3D mechanical profilometer (KLA, Milpitas, CA, USA) was used to determinate the superficial roughness of planar samples in terms of arithmetical mean height of the surface (Sa), maximum height of the surface (Sz), maximum height of valleys (Sv), maximum height of peaks (Sp), kurtosis of height distribution (Sku), skewness of height distribution (Ssk), and root mean square height of the surface (Sq) following ISO 25178. The probe used is 5 µm in diameter, and the scan parameters were: 0.10 mm/s speed and stylus force of 10.0 mg. 3D mapping was performed with 0.025 mm of spacing between profiles. In order to evaluate superficial roughness of nonplanar samples, a JENOPTIK Waveline W10 optic profilometer (JENOPTIK Industrial Metrology Germany GmbH, Villingen-Schwenningen, Germany) following ISO 4287:1999/AC/AC1:2010 [35,36] standard was used. Surface roughness arithmetical mean height (Ra), maximum height of profile (Rz), maximum profile peak height (Rp), and maximum profile valley depth (Rv) values [37] were obtained at the evaluation conditions: λc 0.80 mm, lp 8.000 mm, lr 0.8 mm, lw 0.8 mm, traverse length 8.00 mm, speed 0.50 mm/s, measuring range 400 µm, probe type T3.

The elemental surface composition of the samples in different polishing stages was studied using the X-ray spectrophotometer NITON XL3t (Thermo Fisher Scientific, Waltham, MA, USA). This handheld equipment allows nondestructive testing of a sample by processing the spectrum obtained from X-ray photons and converting the information into elemental analysis. This technique quantifies the amount percent of alloying elements by analyzing a specific area, in this case a 3 mm diameter spot. Three different samples were evaluated. The first one consisted of a raw sample without any treatment. The second one was a smut-containing surface sample (Activation + Step 1), and the last one was a sample treated using a desmutting step (Activation + Step 1 + Step 2 + Desmutting step).

Elemental composition of the smut powder deposited on the aluminum surface formed in the alkaline polishing step was analyzed by Induced Coupled Plasma analysis using Agilent 8900 triple quadrupole ICP-MS (ICP-QQQ) (Agilent, Santa Clara, CA, USA).

### 2.2. Mechanical Properties Experimental Part

**Figure 3:**
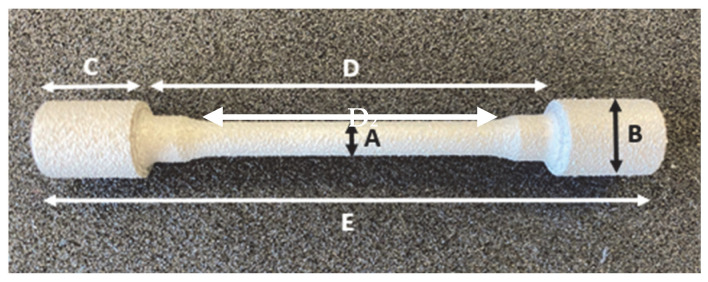
Specimen measurements. A—5.08 mm; B—10.00 mm; C—12.25 mm; D—51.47 mm; D_2_—36.04 mm; E—76.02 mm.

Samples were manufactured with a circular cross section and a central part standardized with a constant section to submit them to tensile testing. The shape of the ending sections of the specimen facilitates fastening into the universal machine jaws (Figure 3).

**Figure 4:**
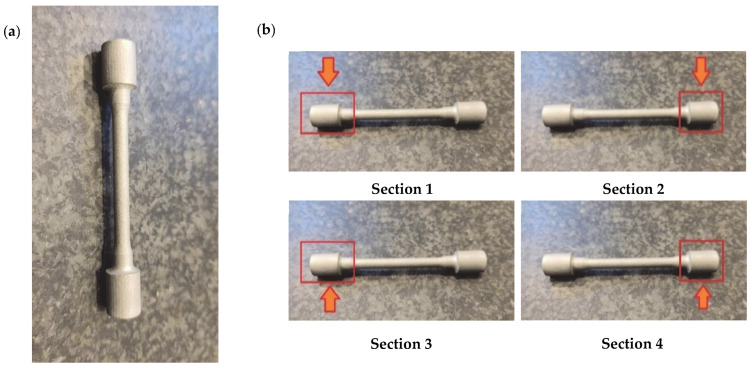
(**a**) AlSi10Mg tensile strength specimen consolidated by SLM technique, (**b**) sections measured by JENOPTIK Waveline W10 optic profilometer.

As these consolidated parts prepared for tensile strength test are not planar, to characterize the roughness of such devices it was necessary to measure it by JENOPTIK Waveline W10 optic profilometer (JENOPTIK Industrial Metrology Germany GmbH, Villingen-Schwenningen, Germany), and 4 sections were established to check the effect before and after the post-treatment in the same area (Figure 4).

The samples were post-treated following the patented process described previously. The roughness was checked again using the same equipment.

Tensile strength of consolidated metal parts was evaluated following UNE EN-ISO 6892-1:2020 [38]. Prior to conducting the test of the specimen, it was ensured that the testing machine was aligned according to the standard ASTM E-1012-19 [39]. The test speed in the elastic zone was 0.0042 mm/mm/min in strain control. The test speed in the plastic zone was 4.32 mm/min in displacement control until fracture. The test was conducted at a room temperature of 23 ± 3 °C and relative humidity of 50 ± 10%. Yield strength was obtained using the offset method according to section 13 of UNE-EN ISO 6892-1:2020 [38] at an offset of 0.2%. The information about the equipment is summarized in Table 1.

## 3. Results and Discussion

**Figure 5:**
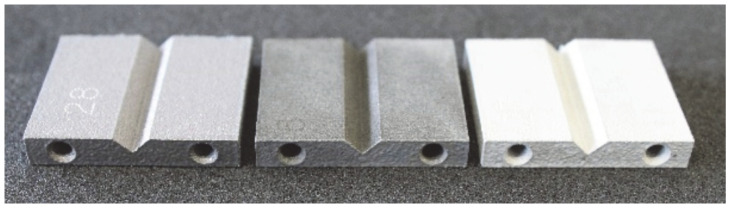
From left to right, a consolidated aluminum metal part as-printed, smut-treated, and a desmutted sample.

The obtained-as-designed SLM consolidated metal parts with pipes and corners, the appearance of an aluminum sample before/after the post-treatment, and a sample which has not been treated with the desmutting step are shown in Figure 5.

In this study, an innovative chemical post-treatment process is described which respects complex geometries, sharps, and performs in inner parts and cavities. Figure 5 shows how edges and corners are maintained, and the diameter of the pipe is slightly increased from 2.85 mm to 3.01 mm after the post-treatment. When the desmutting step is not performed, the sample becomes almost black, and the chemical post-treatment is inefficient. The desmutting step provides brightness thanks to the reduction in the surface roughness. The results of initial roughness measured with a KLA-TENCOR ALPHA STEP D 600 3D mechanical profilometer (KLA, Milpitas, CA, USA) are represented in Table 2.

As can be observed, in order to acquire accurate information on the metal surface roughness, a 3 × 3 mm square area was analyzed. The initial roughness of the consolidated aluminum parts was 20.2 μm Sa, and 162 μm Sz, these two parameters being the ones which provide the most valuable information.

After this initial analysis, the obtained roughness after performing the post-treatment described in Section 2, and repeated 8 times, is shown in Table 3.

After the post-treatment, a significant improvement on AlSi10Mg superficial roughness is demonstrated compared to the initial values. A decrease in Sa, from 20.2 μm (Table 2) to 10.9 μm (Table 3), and from 162 μm (Table 2) to 110 μm (Table 3) for Sz has been observed, meaning a reduction of 46% and 32%, respectively. A matte and smut-free finishing has been obtained after carrying out our novel chemical finishing post-processing on AlSi10Mg metal parts. Common Al/Si/Mg oxide precipitates generated typically during chemical polishing of Al/Si alloys and deposited usually onto their surfaces have not been observed.

Nevertheless, to confirm that our novel chemical polishing post-process removes the smut upper layer, an exhaustive study has been performed on the chemical composition of an aluminum sample on different stages of the polishing method. AlSi10Mg metal parts derived from different stages of chemical polishing method were analyzed using XRF technique. The results obtained are presented in Table 4.

The findings revealed that the, as expected, major constituents in AlSi10Mg alloy are aluminum, silicon, and magnesium. Once the piece was treated with alkaline conditions and smut appeared (giving a black appearance on the metal surface), XRF analysis showed an increase of Si (from 8.684 to 25.042%) and Mg (from 0.587 to 0.858%). The black powder formed, then, was a result of a deposition of Mg(OH)_2_ and SiO_2_·xH_2_O on the aluminum surface [26]. Al detection decreases from 90.095% of the raw sample, which corresponds to mainly aluminum oxide, to 73.512% measured on the sample with smut. This is because smut layer is deposited on top of the metal surface and interferes with Al identification. Once smut is removed, Al is again revealed (90.940%).

Finally, smut formed in the alkaline treatment step was removed from the AlSi10Mg samples using the acid desmutting step liquid and in order to submit it to analysis. The presence of elements was measured by ICP-MS using a semiquantitative method (“SemiQuant”) (Agilent 8900 triple quadrupole ICP-MS (ICP-QQQ),Agilent, Santa Clara, CA, USA) in which both the composition and approximate (typically better than ±30%) concentration of a sample can be measured in a single analysis without any previous knowledge of the sample [40]. The results are shown in Table 5.

This table shows that the main component of the black powder is Si (83%), so this explains that most of the product formed on the alkaline treatment is a component formed by silicon (SiO_2_·xH_2_O). These results are correlated with the XRF results shown in Table 4 that also explain that the main element present in smut is Si (25.042%). The subproducts formed may be other oxides from magnesium and iron (elements present in the alloy). Aluminum which comes from the metal piece is also detected because the smut powder extraction is performed by etching the piece to undergo ICP-MS analysis.

Illustrated also in Table 4, Si values were reduced to initial numbers when the sample was treated with the desmutting step (from 25.042 to 8.086%), which was related with the elimination of the oxides formed during alkaline treatment. Furthermore, Mg percentage is reduced (from 0.858% to 0.421%), meaning that desmutting treatment not only eliminates silicon oxides formed but also magnesium ones.

### 3.1. Sample Preparation for Tensile Strength Test

As the investigated post-process consists of a dip treatment, the liquid could seep through the porosity given in metal parts when manufactured by SLM manufacturing routes. To prove that the novel chemical polishing method does not have any influence on bulk properties, and so that the chemical components from different formulations do not further attack the interior of the metal, the samples manufactured with a circular cross section were post-treated and submitted to tensile testing.

The samples were post-treated following the patented process described previously, obtaining a modification of 15% from the initial measurements of the samples (Table 6); Section A goes from 5.08 to 4.25 cm.

Also, samples were weighed before and after the treatment. The initial average weight corresponding to the samples is 7.55 g and the average final weight is 6.96 g, thus obtaining an average mass decrease of 7.77%. The obtained roughness results for consolidated samples before (Table 7) and after the post-processing (Table 8) are represented below.

The roughness results obtained after the samples were post-processed, as can be observed in Table 8, show an improvement in roughness values (Ra goes from average 13.98 to 9.38 µm and Rz from 67.41 to 42.01 µm), also represented in terms of percent reduction in Table 9 (reduction of 40% in Ra and 38% in Rz). Repeatability is demonstrated by means of standard deviation, analyzing Ra value in average reduction is only 0.31 µm and Rz standard deviation is higher (1.28 µm) due to the fact that the height of peaks and valleys is dissimilar from the beginning. Additionally, peaks and valleys heights, represented by Rp and Rv, respectively (total average reduction of 35 and 41%), also decrease compared to the initial metal part.

### 3.2. Tensile Strength Test According to UNE EN-ISO 6892-1:2020 [38]

Once the samples were polished following the novel chemical polishing process, tensile strength tests were evaluated according to UNE EN-ISO 6892-1:2020 [38] and compared with nontreated samples. The results are shown in Table 10, and the elongation curve of each sample tested is represented in Appendix A.

The results obtained indicate that final mechanical properties of chemically polished AlSi10Mg parts are maintained from initial values corresponding to nontreated AlSi10Mg parts (Table 10). Young modulus, yield strength, and tensile strength provide data on mechanical behavior directly. The first one, also known as elastic modulus, provides information about the stiffness of the material. There are no differences between nontreated and treated specimens’ values, which go from 61.1 ± 1.5 to 62.2 ± 2.6 GPa.

However, despite the differences not being very significant, the comparison between yield strength and tensile strength shows a trend which provides information about other potential advantages of polishing apart from finishing (i.e., reduction in surface roughness) and aesthetics (i.e., optical aspect of AM part surfaces). Treated samples have major values of both properties, which are shown in Table 10: yield strength is increased by 4% (average values go from 236.8 to 246.7 MPa), while tensile strength by about 3% on average (from 371.9 to 382.5 MPa). During the manufacturing process, parameters such as printing direction, laser parameters, and initial powder properties can cause cracks. These cracks increase the risk of mechanical failures and premature fractures during the operation of parts or during a mechanical test [41]. An inefficient post-process could have increased the depth of these microcracks leading to a negative impact in yield strength and tensile strength. On the contrary, the obtained results demonstrate that the present polishing process is responsible for mitigating this issue by removing the upper layer of the metal part, and thus these inclusions and microcracks present on the metal surface serve as failure initiation points. Parts produced from SLS technique are built up in a layer-by-layer manner and their properties depend on the direction in which they are deposited. Specimens of this report are produced on Z-direction (vertical) and cause samples to be more sensitive to crack initiation due to the presence of more borderline porosity [42].

## 4. Conclusions

The surface roughness of AlSi10Mg metal parts manufactured by SLM technique can be notably decreased by utilizing a novel environmentally friendly chemical polishing post-process. The final roughness requirements are achieved through a loop treatment that blends alkaline and acidic baths. The roughness parameters can be decreased up to 40% thanks to the described chemical polishing. The surface toughness values have been confirmed through an extensive study of the surface roughness by means of two different techniques: mechanical and optical profilometry. The process has demonstrated repeatability and reproducibility, with multiple samples tested, as is described in the report. Moreover, the new chemical polishing method’s desmutting efficiency has been evaluated. XRF analysis demonstrates that AlSi10Mg alloy maintains its alloy elements after the post-treatment, and so, the typical precipitates from smut, which are Si and Mg, are removed thanks to the desmutting step. This methodology has been further supported by the ICP-MS technique that analyzed the removed outer layer. Finally, the tensile strength of consolidated pre- and post-treated AlSi10Mg metal components revealed that the new chemical surface treatment introduced in this study does not have detrimental effects on the bulk material. In fact, there has been a slight improvement due to a reduction in defects found on the metal surface, which act as failure initiation points. It is evident that this new chemical treatment can be beneficial for enhancing the overall quality of AlSi10Mg metal parts.

A technique has been developed to polish the surface of AlSi10Mg, which improves superficial roughness without compromising its mechanical properties. This will help to consolidate the implementation of additive manufacturing technologies in the industry, by providing not only an aesthetic finish but also the surface functionalities and overall performance of the printed device will be enhanced. The novel methodology outlined in this paper is ideal for use on printed parts with complex geometries, cavities, and pipes. This feature sets it apart from conventional polishing processes (both mechanical and electrochemical). The development of post-processing solutions unlocks fresh applications that were previously unattainable, spanning from healthcare to industrial machinery.

## Figures and Tables

**Table 1:** Equipment used in tensile strength test.

Equipment	Model
Universal Testing Machine “100 kN”	MTS 370.25
Extensometer	MTS 632.11F-90
Digital Calliper	MITUTOYO CD-15APX

**Table 2:** Roughness results in terms of quadratic area of AlSi10Mg consolidated metal parts, and 2D/3D surface profiles.

Parameters	Sq	Ssk	Sku	Sp	Sv	Sz	Sa
**Values (µm)**	25.5	−0.13	3.00	79.4	82.2	162	20.2
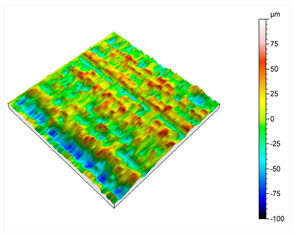				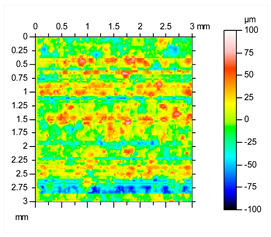			

**Table 3:** Roughness results in terms of quadratic area of AlSi10Mg post-processed metal parts, and 2D/3D surface profiles.

Parameters	Sq	Ssk	Sku	Sp	Sv	Sz	Sa
**Values (µm)**	13.9	−0.287	3.46	43.8	66.1	110	10.9
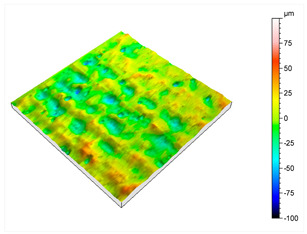			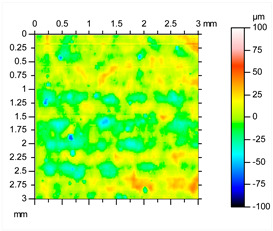				

**Table 4:** Element sorting (%) by XRF technique on different aluminum samples.

Element (%)	Raw Sample	Sample with Smut	Desmutted Sample
Si	8.684 ± 0.115	25.042 ± 0.179	8.086 ± 0.108
Mg	0.587 ± 0.174	0.858 ± 0.173	0.421 ± 0.201
Fe	0.244 ± 0.007	0.250 ± 0.007	0.222 ± 0.007
Zn	0.005 ± 0.001	0.005 ± 0.001	0.005 ± 0.001
Ni	0.012 ± 0.003	0.014 ± 0.003	0.012 ± 0.003
Cu	0.004 ± 0.001	0.005 ± 0.001	0.004 ± 0.001
Al	90.095 ± 0.144	73.512 ± 0.127	90.940 ± 0.139

**Table 5:** Approximate elemental composition of smut obtained in the alkaline treatment step.

Element	Si	Mg	Fe	Al
**Composition (%)**	83	1	2	14

**Table 6:** Tensile strength samples section before and after polishing. Sections corresponding to Figure 3.

(cm)	Section A	Section B	Section C	Section D	Section D2	Section E
Before polishing	5.08	10.00	12.25	51.47	36.04	76.02
After polishing	4.25	9.80	12.18	51.41	35.96	75.96

**Table 7:** Optic profilometer average roughness measurement values obtained from 3 replicates before post-processing. (**a**) Average results per section, (**b**) average roughness results of the samples.

(**a**)	**Section 1**	**Section 2**	**Section 3**	**Section 4**
	**Average**	**Average**	**Average**	**Average**
Ra (µm)	15.52 ± 1.69	14.33 ± 1.23	13.48 ± 0.82	12.60 ± 0.81
Rz (µm)	72.14 ± 5.80	66.48 ± 4.12	67.68 ± 1.96	63.34 ± 4.66
Rp (µm)	36.00 ± 4.46	32.69 ± 1.95	35.90 ± 1.58	30.99 ± 3.63
Rv (µm)	36.13 ± 1.61	33.79 ± 3.09	31.78 ± 0.87	32.35 ± 1.06
(**b**)	**Global Average**			
Ra (µm)	13.98 ± 0.65			
Rz (µm)	67.41 ± 2.26			
Rp (µm)	33.89 ± 1.61			
Rv (µm)	33.51 ± 1.02			

**Table 8:** Optic profilometer average roughness measurement values obtained from 3 replicates after post-processing. (**a**) Average results per section, (**b**) average roughness results of the samples.

(**a**)	**Section 1**	**Section 2**	**Section 3**	**Section 4**
	**Average**	**Average**	**Average**	**Average**
Ra (µm)	8.07 ± 0.18	9.08 ± 0.37	7.84 ± 0.24	8.52 ± 0.80
Rz (µm)	41.86 ± 2.62	43.26 ± 3.54	41.33 ± 3.19	41.58 ± 2.54
Rp (µm)	21.08 ± 2.17	22.56 ± 2.89	22.05 ± 1.72	22.61 ± 1.35
Rv (µm)	20.79 ± 0.44	20.70 ± 0.65	19.28 ± 1.47	18.97 ± 1.35
(**b**)	**Global Average**			
Ra (µm)	8.38 ± 0.31			
Rz (µm)	42.01 ± 1.28			
Rp (µm)	22.07 ± 0.81			
Rv (µm)	19.93 ± 0.65			

**Table 9:** Total average reduction of roughness values after AlSi10Mg parts post-treatment.

	Roughness Reduction (µm)	Reduction (%)
Ra	5.6 ± 0.31	40
Rz	35.4 ± 1.28	38
Rp	11.8 ± 0.81	35
Rv	13.6 ± 0.65	41

**Table 10:** Numeric tensile strength results.

Treatment	Specimen	Young Modulus (Gpa)	Yield Strength Re (Mpa)	Tensile Strength Rm (Mpa)
Nontreated	Al 1	62.7	238	373
	Al 2	60.0	237	364
	Al 3	60.4	236	378
	**Average**	**61.1 ± 1.5**	**236.8 ± 0.8**	**371.9 ± 7.1**
Treated	Al 1	59.3	242	388
	Al 2	62.9	249	378
	Al 3	64.5	249	382
	**Average**	**62.2 ± 2.6**	**246.7 ± 4.0**	**382.5 ± 5.0**

**Figure A1:**
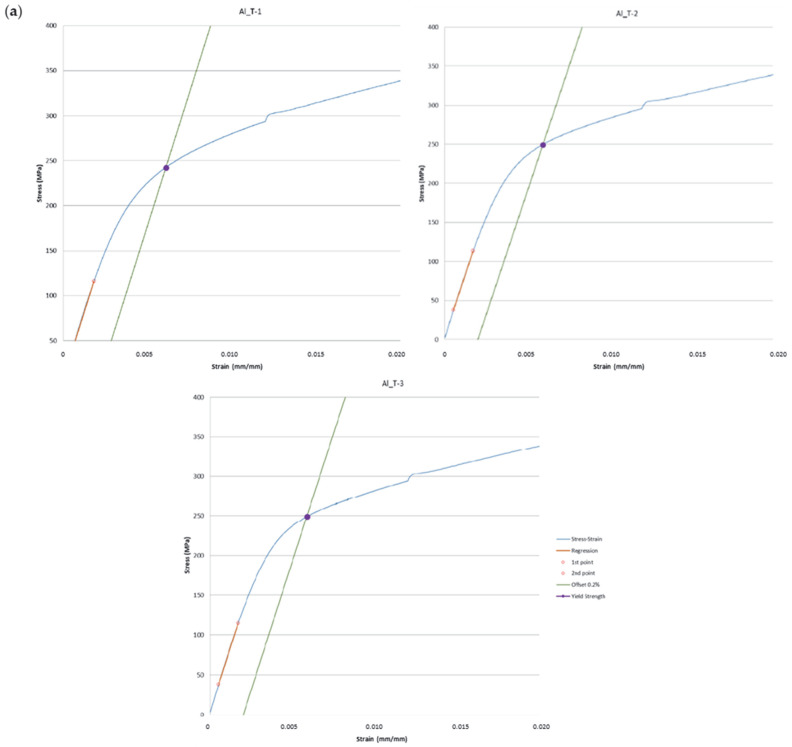

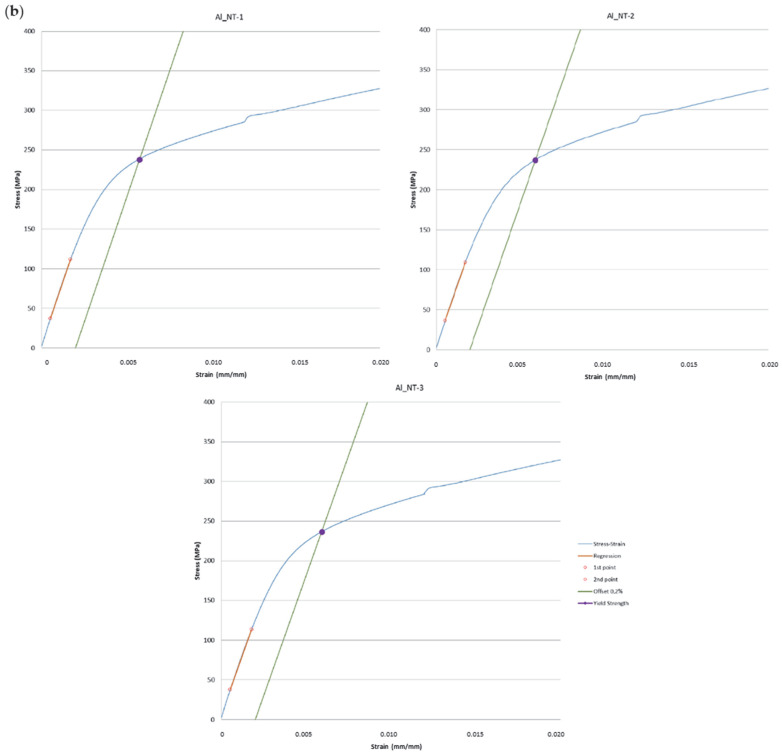
Elongation curves from the stress–strain tests corresponding to the treated aluminum samples (**a**) compared with the nontreated ones (**b**).

## Data Availability

Not applicable.

## References

[1] Zhang J., Song B., Qinsong W., Bourell D., Yusheng S. (2019). A Review of Selective Laser Melting of Aluminum Alloys: Processing, Microstructure, Property and Developing Trends. J. Mater. Sci. Technol..

[2] Olakanmi E.O., Cochrane R.F., Dalgarno K.W. (2015). A Review on Selective Laser Sintering/Melting (SLS/SLM) of Aluminium Alloy Powders: Processing, Microstructure, and Properties. Prog. Mater. Sci..

[3] Mondolfo L.F. (2013). Aluminum Alloys: Structure and Properties.

[4] Polmear I., StJohn D., Nie J.-F., Qian M. (2017). Light Alloys: Metallurgy of the Light Metals.

[5] Murr L.E. (2020). Metallurgy Principles Applied to Powder Bed Fusion 3D Printing/Additive Manufacturing of Personalized and Optimized Metal and Alloy Biomedical Implants: An Overview. J. Mater. Res. Technol..

[6] Mostafaei A., Zhao C., He Y., Reza Ghiaasiaan S., Shi B., Shao S., Shamsaei N., Wu Z., Kouraytem N., Sun T. (2022). Defects and Anomalies in Powder Bed Fusion Metal Additive Manufacturing. Curr. Opin. Solid State Mater. Sci..

[7] Liu W., Wei H., Liu A., Zhang Y. (2022). Multi-Index Co-Evaluation of Metal Laser Direct Deposition: An Investigation of Energy Input Effect on Energy Efficiency and Mechanical Properties of 316l Parts. J. Manuf. Process..

[8] Lee J.-Y., An J., Chua C.K. (2017). Fundamentals and Applications of 3D Printing for Novel Materials. Appl. Mater. Today.

[9] Gao W., Zhang Y., Ramanujan D., Ramani K., Chen Y., Williams C.B., Wang C.C.L., Shin Y.C., Zhang S., Zavattieri P.D. (2015). The Status, Challenges, and Future of Additive Manufacturing in Engineering. Comput.-Aided Des..

[10] Shad A., Stache R., Rütjes A. (2021). Effects of Fumed Silica Flow Aids on Flowability and Packing of Metal Powders Used in Binder-Jetting Additive Manufacturing Process. Mater. Des..

[11] Prashar G., Vasudev H. (2021). A Comprehensive Review on Sustainable Cold Spray Additive Manufacturing: State of the Art, Challenges and Future Challenges. J. Clean. Prod..

[12] Liu Y., Yang Y., Mai S., Wang D., Song C. (2015). Investigation into Spatter Behavior during Selective Laser Melting of AISI 316L Stainless Steel Powder. Mater. Des..

[13] Leon A., Aghion E. (2017). Effect of Surface Roughness on Corrosion Fatigue Performance of AlSi10Mg Alloy Produced by Selective Laser Melting (SLM). Mater. Charact..

[14] Brandl E., Heckenberger U., Holzinger V., Buchbinder D. (2012). Additive Manufactured AlSi10Mg Samples Using Selective Laser Melting (SLM): Microstructure, High Cycle Fatigue, and Fracture Behavior. Mater. Des..

[15] Altıparmak S.C., Yardley V.A., Shi Z., Lin J. (2020). Challenges in Additive Manufacturing of High-Strength Aluminium Alloys and Current Developments in Hybrid Additive Manufacturing. Int. J. Light. Mater. Manuf..

[16] Clancy K. (2022). Annual Additive Manufacturing 3D Post-Printing Survey Results. Javelin 3d Solut..

[17] Malakizadi A., Mallipeddi D., Dadbakhsh S., M’Saoubi R., Ktajnik P. (2022). Post-Processing of Additively Manufactured Metallic Alloys—A Review. Int. J. Mach. Tools Manuf..

[18] Hu Z., Wan L., Lü S., Zhu P., Wu S. (2014). Research on the Microstructure, Fatigue and Corrosion Behavior of Permanent Mold and Die Cast Aluminum Alloy. Mater. Des..

[19] Meseguer T., Soldi L., Calvet M., Domenech A., Dominguez E. Method for Polishing Parts Made of Aluminum. WO 2021/094641 A1, 15/11/2019.

[20] Sood A., Mullany B. (2021). Advanced Surface Analysis to Identify Media-Workpiece Contact Modes in a Vibratory Finishing Processes. Procedia Manuf..

[21] Metal Parts Vibrating and Dry Vibration Polishing. https://www.coniex.com/en/vibration/.

[22] Rotary Vibrators—Rösler Oberflächentechnik GmbH. https://uk.rosler.com/uk-en/products/mass-finishing/rotary-vibrators/.

[23] Uhlmann E., Eulitz A. (2018). Influence of Ceramic Media Composition on Material Removal in Vibratory Finishing. Procedia CIRP.

[24] Bhaduri D., Penchev P., Dimov S., Essa K., Carter L.N., Pruncu C.I., Jiang J., Pullini D. (2020). On the Surface Integrity of Additive Manufactured and Post-Processed AlSi10Mg Parts. Procedia CIRP.

[25] Mahmood M.A., Chioibasu D., Ur Rehman A., Mihai S., Popescu A.C. (2022). Post-Processing Techniques to Enhance the Quality of Metallic Parts Produced by Additive Manufacturing. Metals.

[26] Wilson B.P., Dotremont A., Biesemans M., Willem R., Campestrini P., Terryn H. (2008). Effect of Additives on Smut-Layer Formation and Pitting during Aluminum Etching in Hydrochloric Acid. J. Electrochem. Soc..

[27] Basha M.M., Basha S.M., Jain V.K., Sankar M.R. (2022). State of the Art on Chemical and Electrochemical Based Finishing Processes for Additive Manufactured Features. Addit. Manuf..

[28] Tyagi P., Goulet T., Riso C., Stephenson R., Chuenprateep N., Schlitzer J., Benton C., Garcia-Moreno F. (2019). Reducing the Roughness of Internal Surface of an Additive Manufacturing Produced 316 Steel Component by Chempolishing and Electropolishing. Addit. Manuf..

[29] Chen G.S., Wan K.-C., Gao M., Wei R.P., Flournoy T.H. (1996). Transition from Pitting to Fatigue Crack Growth—Modeling of Corrosion Fatigue Crack Nucleation in a 2024-T3 Aluminum Alloy. Mater. Sci. Eng. A.

[30] Jin Z., Cai C., Hashimoto T., Yuan Y., Kang D., Hunter J., Zhou X. (2020). Alkaline Etching and Desmutting of Aluminium Alloy: The Behaviour of Mg2Si Particles. J. Alloys Compd..

[31] Mesicek J., Ma Q.-P., Hajnys J., Zelinka J., Pagac M., Petru J., Mizera O. (2021). Abrasive Surface Finishing on SLM 316L Parts Fabricated with Recycled Powder. Appl. Sci..

[32] Maleki E., Bagherifard S., Unal O., Sabouri F., Bandini M., Guagliano M. (2022). Effects of Different Mechanical and Chemical Surface Post-Treatments on Mechanical and Surface Properties of as-Built Laser Powder Bed Fusion AlSi10Mg. Surf. Coat. Technol..

[33] Limbasiya N., Jain A., Soni H., Wankhede V., Krolczyk G., Sahlot P. (2022). A Comprehensive Review on the Effect of Process Parameters and Post-Process Treatments on Microstructure and Mechanical Properties of Selective Laser Melting of AlSi10Mg. J. Mater. Res. Technol..

[34] Data Sheet: AlSi10Mg-0403 (400 W) Powder for Additive Manufacturing. https://www.renishaw.com/resourcecentre/en/details/data-sheet-alsi10mg-0403-400-w-powder-for-additive-manufacturing--73122.

[35] Geometrical Product Specifications (GPS)—Surface Texture: Profile Method—Terms, Definitions and Surface Texture Parameters—Amendment 1: Peak Count Number (ISO 4287:1997/Amd 1:2009). https://www.une.org/encuentra-tu-norma/busca-tu-norma/norma?c=norma-une-en-iso-4287-1999-a1-2010-n0046045.

[36] Geometrical Product Specifications (GPS)—Surface Texture: Profile Method—Terms, Definitions and Surface Texture Parameters (ISO 4287:1997/Cor 1:1998/Cor 2:2005). https://www.une.org/encuentra-tu-norma/busca-tu-norma/norma/?c=N0045298.

[37] (2020). Surface Texture: Surface Roughness, Waviness, and Lay.

[38] Metallic Materials—Tensile Testing—Part 1: Method of Test at Room Temperature (ISO 6892-1:2019). https://www.une.org/encuentra-tu-norma/busca-tu-norma/norma/?c=N0064441.

[39] Standard Practice for Verification of Testing Frame and Specimen Alignment Under Tensile and Compressive Axial Force Application. https://www.astm.org/e1012-19.html.

[40] Wilbur S. (2004). Applications of ICP-MS in Homeland Security.

[41] Galy C., Le Guen E., Lacoste E., Arvieu C. (2018). Main Defects Observed in Aluminum Alloy Parts Produced by SLM: From Causes to Consequences. Addit. Manuf..

[42] Kempen K., Thijs L., Van Humbeeck J., Kruth J.-P. (2012). Mechanical Properties of AlSi10Mg Produced by Selective Laser Melting. Phys. Procedia.

